# Profiling the variability and inequity in the residential environment in Cyprus according to citizens’ ratings: a cross-sectional internet-based “Place Standard” survey

**DOI:** 10.1186/s12889-022-12706-y

**Published:** 2022-02-09

**Authors:** Daphne Kleopa, Andrie Panayiotou, Christiana Kouta, Chrystalla Kaiafa, Nicos Middleton

**Affiliations:** 1 Department of Nursing, School of Health Sciences, Cyprus University of Technology, Limassol, Cyprus; 2 Cyprus International Institute for Environmental and Public Health, School of Health Sciences, Cyprus University of Technology, Limassol, Cyprus; 3 WHO Healthy Cities Cyprus Network Coordinating Office, Cyprus Ministry of Health, Nicosia, Cyprus

**Keywords:** Place, Place standard, Community assessment, Neighbourhood environment, Perception survey, Environmental inequity, Social gradient, Measurement tool, Validity

## Abstract

**Background:**

The “Place Standard Tool” (PST) offers a practical framework for structuring conversations about physical and social dimensions of Place which impact on health and well-being. The aim of this study was to survey citizens’ perceptions of Place across diverse settings in Cyprus. While the PST has been extensively used in the context of community engagement, its properties as a measurement tool haven’t been explored.

**Methods:**

An open call was addressed to citizens to rate their neighbourhood environment across the 14 PST items (1: large to 7: little room for improvement). Exploratory factor, cluster and regression analyses were used to explore the dimensionality of the scale, depict neighbourhood profiles and explore differences in ratings according to socio-demographic characteristics, area-level census indicators and residents’ assessment of neighbourhood social position (10-step ladder).

**Results:**

With the exception of safety (M = 4.4, SD = 1.7), 492 participants (mean age 42, 50% residents for > 10 years) from 254 postcodes (21.7% islandwide) did not rate other features favourably, with lowest scores for “influence and sense of control” and “public transport”. A stepwise pattern of dissatisfaction was observed along the social position continuum both for features rated less as well as more favourably (e.g. social contact). For instance, among participants who placed their neighbourhood at the three top steps of the ladder, 48.8% gave a low rating for “influence and sense of control”, while the equivalent figure was 81.0% at the bottom three steps (OR = 4.5, 95% CI 2.3, 8.6). A clear dimensionality of Built (6 items, Cronbach’s α = 0.798), Physical (3 items, α = 0.765), Social (2 items, α = 0.749) and Service (3 items, α = 0.58) environment was identified. A social gradient was evident according to census measures of socio-economic disadvantage (e.g. pre-1980 housing, single-parent households) with larger differences in terms of the built than the social environment.

**Conclusions:**

The study profiled the variability and documented the inequity in the health-related neighbourhood environment across Cypriot communities. The readily interpretable dimensionality of the scale supports its construct validity, allowing calculation of composite scores. The PST can be used as measurement tool in research as well as public health practice to advocate for neighbourhood initiatives which support and enhance citizens’ participation.

**Supplementary Information:**

The online version contains supplementary material available at 10.1186/s12889-022-12706-y.

## Introduction

The community assessment toolbox comprises of a variety of methods for profiling the health-related residential environment [1], each with strengths as well as shortcomings. This includes the use of census or other administrative data (e.g. indices of socio-economic deprivation, crime statistics, etc.), GIS or remote sensing measures (e.g. air pollution, green space, destinations, accessibility etc.), neighbourhood audits (e.g. food environment, walkability, physical disorder or other microscale features of the neighbourhood environment), residents’ perception surveys (e.g. quality or problems of the neighbourhood environment in relation to well-being), qualitative and mixed-methods (e.g. in-depth understanding of residents’ lived experience) and, increasingly, participatory approaches, such as photo mapping and photovoice action research, which also target neighbourhood empowerment and advocacy [2–5]. These methods can be employed alone or in combination depending on the purpose (e.g. in the context of Public Health policy or research), main focus of enquiry (e.g. physical and/or social aspects of place) and objectives (e.g. neighbourhood profile vs citizens’ views, needs and perceptions).

While neighbourhood audits allow to ‘objectively’ (i.e. free of residents’ perceptions) assess microscale features of the residential environment [6, 7], studies often report a mismatch between observer-rated neighbourhood features and residents’ perceptions [8, 9]. With regard to surveying residents’ perceptions on the health-related residential environment, both opportunistic use of a set of items and purposefully-designed tools have been described in the literature, spanning the academic fields of urban planning, environmental psychology and public health. Some are generic i.e. tapping on several domains of the residential environment [10, 11] while others are outcome-, feature-, or population-group-specific; for instance, tapping on the determinants of specific health outcomes of interest, such as cardiovascular health [12, 13], or, in-depth description of specific features of place, such as walkability or other characteristics which may promote physical activity [14], or even targeted on the needs of specific population groups, such as physical activity among young people [15].

The “Place Standard Tool” (PST), developed in Scotland, is not a measurement tool per se, but a practical participatory action tool that, to quote “*translates complex health and place-making relationships into a simple set of questions about place – meaningful to communities, and public, private and third sectors*” [16]. Hes et al. (2021) mentions that there are over 75 such frameworks, indices, guidelines and engagements tools used in Place evaluation [17], concluding that it is not a question of which tool or method is best, but about holistically evaluating, while fostering in the process, the relationships between people and places.

The PST has been used across numerous local authorities in Scotland reaching thousands of citizens within the first year of roll-out alone [18]. It is highly transferrable across settings and, through the European Network for WHO Healthy Cities, the Place Standard has also been implemented in several case studies across at least 14 international settings, including the Netherlands, Spain, North Macedonia and Germany.

In Cyprus, there is not a strong tradition of community engagement in the context of place-making while “place” is not a strong feature in the public health agenda in general. Thus, community assessment studies are not prominent either in the context of policy and practice or epidemiological and community health research [19]. To date, only a handful of studies have profiled the contextual characteristics of Cypriot communities and documented the social gradient in health [20, 21]. Driven in parallel by the lack of generally accepted composite indices of socio-economic disadvantage, these studies employed “traditional” census-based approaches of exploring the nationwide or city-level spatial patterning of socio-geographical inequalities in health.

In 2019, the Cyprus National Network of WHO Healthy Cities was founded with the participation of 11 municipalities. Two municipalities have since expressed their interest in applying for certification in the Phase VII (2019–2024) programme. The Healthy Cities movement has been influential at the local level in setting up systems across a network of cities in order to produce or improve locally-generated data to profile a city’s people and places, with an increasing integration of methodological approaches [22]. One of the initial requirements is the preparation of a City’s Health Profile, which would consider a range of objective as well as subjective indicators of health and health determinants, including Place, with a focus on environmental inequity.

The Place Standard appears promising in the context of community engagement and advocacy due to its simplicity, yet comprehensiveness, in covering several health-related dimensions of place. Among its strengths is the visual representation of assets and weakness of place (in the form of a radial plot) and its flexibility in its application by individuals or groups alike. With roots in community engagement, it has been more likely to be used as a practice and policy tool, rather than a scoring tool as part of a wider community health research study. While there are many implementation case study reports [23], surprisingly, only a small number of articles reporting the use of PST were identified in the peer-reviewed literature, specifically in Woodside, Glasgow, Scotland [24], Skopje, North Macedonia [25] and Nicosia, Cyprus [26]. These studies differ in terms of their aims, study design and/or field of enquiry. For example, the case study from Cyprus [26] sampled a convenience sample of five adults aged 56–80 years across a purposive sample of five residential postcodes in Nicosia with differing urban planning features. Furthermore, a study in Moscow, Russia [27] discussed the use of Place Standard alongside other tools and approaches, focusing on the implications of different frameworks involving conventional versus digital tools for community engagement. None of these studies have fully explored the construct validity or other metric properties of the scale. Furthermore, the extent to which the Place Standard captures the variability and social gradient in neighbourhood environment across a set of heterogeneous communities has not been explored.

The aim of this study was to (a) survey citizens’ perceptions on the quality of neighbourhood environment and profile Place along the dimensions of the Place Standard across diverse settings in Cyprus, (b) explore the reliability and construct validity of the scale in terms of capturing distinct dimensions of Place, (c) investigate the association of scale and sub-scale PCT score ratings with socio-demographic characteristics and census-based area indicators, and (d) depict the social gradient in residential environment against residents’ subjective assessment of their neighbourhood’s social position.

## Methods

Under the auspices of the Healthy Cities Office of the Cyprus Ministry of Health, an internet-based survey was performed between April–May 2019. It involved an open call to citizens to rate their neighbourhood environment along the 14 dimensions of the Place Standard Tool. Alongside the motto “*if we don’t measure it, we won’t improve it*”, the study material featured for promotional purposes an original street art image found on a wall in old Nicosia’s historic centre, depicting a café-like scene in a town square where people of all ages and backgrounds appear to have come together in a common task (see Additional file 1).

The study formed part of the wider CyNOTes (Cyprus Neighbourhoods Observational Tool for auditing community environments) study [28], which aims to develop, test and validate a Systematic Social Observation tool for auditing microscale features of Cypriot urban neighbourhoods. Part of the validation process involves a door-to-door survey in a sample of neighbourhoods in the city of Limassol in order to assess the observer-based ratings against residents’ perceptions of neighbourhood environment [29]. For the door-to-door survey, the Place Standard questionnaire was adopted due to its clarity and brevity. The purpose of the internet-based Place Standard survey reported here was to pilot use the tool, assess its metric properties and provide a reference against which the profile of CyNOTes neighbourhoods can be compared to. Other than widening participation beyond the sample of audited neighbourhoods, the collaboration with the Healthy Cities Office provided an opportunity to encourage a public dialogue about the importance of Place for health and well-being.

### Place Standard Tool

The Place Standard Tool (PST) provides a framework to structure conversations about Place in a holistic view but along a measurable set of dimensions. It was developed by NHS Scotland in partnership with the Scottish Government, Architecture and Design Scotland and Glasgow City Council. More than just a profiling tool, the PCT’s main goal is to prompt and encourage an inclusive dialogue among stakeholders in order to identify assets and recourses as well as challenges, pinpoint to areas for improvement and assist in setting priorities by consensus. Launched in 2015, it has since been used extensively in Scotland to engage with communities, develop a shared understanding of priorities and actions and shape collaborative decisions. Τhe tool can be used to assess different types and sizes of Places by individuals or groups. Available in multiple forms, including booklet, interactive and web version [30], the tool allows flexibility. It can be used in print or digital form, in the context of surveys or focus groups, in formal settings or in walk-abouts.

The PST contains a series of 14 items, each addressing a different dimension of Place, covering both physical as well as social aspects that can impact on health and well-being. Namely, these are: Moving Around, Public Transport, Traffic & Parking, Streets & Spaces, Natural space, Play & Recreation, Facilities & Amenities, Work & Local economy, Housing & Community, Social contact, Identity & Belonging, Feeling safe, Care & Maintenance, Influence & Sense of control. The Place Standard is designed to allow responders to provide a quantitative assessment of each dimension on a scale from 1 = large room for improvement to 7 = little room from improvement as well as a qualitative assessment in the form of free text to identify what shaped the particular rating. For each of the 14 items, prompts in the form of supplementary questions describe various aspects related to that domain for the responder’s consideration. A nice feature of the tool is that the quantitative scores are displayed on a compass graph (radial plot) to provide an overall profile of Place and thus illustrating visually strengths (approaching the outward circle with highest possible score of 7) and weaknesses (closer to the inner circle with lowest score of 1).

### Translation to Greek

Permission to use the Place Standard was obtained by the developers. The tool was translated and adapted into Greek from the original English using a forward and backward translation process. The full booklet version, including the introductory information and instructions for completion, was translated into Greek by two of the authors independently of each other (NM and DK). After discussing and consolidating any discrepancies, a single Greek version was back-translated into English independently by the other two authors (AP and CK). Any issues were resolved by consensus with a focus on semantic equivalence. During the process, we were contacted by the Place Standard team with the request to collaborate with another team based in Cyprus (Cyprus Energy Agency) which had also used the Place Standard in the context of a Nicosia-based neighbourhood initiative. The two Greek translations were remarkably close and any small discrepancies were discussed and resolved through consensus between the teams. This resulted in the final version which was forwarded to the original team for future reference along with detailed explanations and justifications with regard to any adaptations deemed necessary, all of which were minor and did not result in any substantial differences from the original English version.

The most challenging aspect of the translation was not the content per se but the key terms “Place” and “Standard”, alone or in combination, since different words take a different meaning depending on the context used. This does not seem to be particular to Greek and it has been reported in the context of the translation of the tool into other languages. For instance, the chosen term in Greek to represent “standard” (i.e. “πρότυπη”) is the term used in Epidemiology for the process of standardization of rates against a “standard population”, but it can also take the meaning of “indicative example”, “template” or even “ideal” depending on context. Similarly, the Greek term for ‘Place’ (i.e. τόπος) has an awkward sound when used alone, as it is more commonly followed by “of birth”, “of residence” or “of work”, whereas when used with “my” it carries the meaning of a one’s “homeland” whether referring to a village or the country. In the online survey, the term “neighbourhood” was preferred since this is readily understandable and better aligned with the purposes of the particular study. Nevertheless, for the standard Greek version, both terms (i.e. neighbourhood and place) where used as necessary to convey that the tool is not restricted to rating neighbourhoods but can be used for any Place, including public spaces or whole communities.

### Adaptation to an online format

The Place Standard Tool was adapted to an online format using Google Forms. The first page provided information about the aims of the study, a description of the questionnaire structure (main and prompt questions) and instructions for completion. For the purpose of this study, all prompt questions were included in the online version, however the option for open-ended responses was not provided. Similarly, while the booklet version of the Greek translation of the Place Standard contains all features, including the identification of priorities and actions, these were not included in the online version.

In terms of the structure, each domain of the Place Standard was presented on a separate page. At the top of each page there was a short introduction, as per the original Place Standard, on why this dimension of Place is important, followed by the main question featuring a response scale from 1–7 with characterizations at either end whereby 1 = large room for improvement and 7 = little room for improvement. In each case, the responder was asked to consider a range of aspects related to this dimension before providing a rating, again as per the original Place Standard, by providing prompt questions. Each question featured a characteristic Cyprus-specific picture of the dimension in question to increase familiarization. Both public-domain pictures (e.g. a picture of the identifiable Municipality of Limassol building for “Influence and sense of Control”) as well as original photographs were used as fit for purpose. For instance, “social contact” was portrayed by a photo of two elderly men playing backgammon on a front house veranda; which is a more common form of social activity in Cyprus in certain age-groups than meeting people in formal or informal community settings.

### Socio-demographic information and subjective neighbourhood social position

Participants were requested to provide basic socio-demographic information, including gender, age, nationality, educational attainment, marital and employment status and whether they had any financial difficulties e.g. paying bills in the past 12 months. Participants also provided information regarding the size of household, house tenure status, house type, length of residence in the current address. The 4-digit postcode was recorded along with the name of municipality (metropolitan areas) or community (rural areas) and district of residence to allow validation. Postcodes are the smallest unit of geographical aggregation for the census purposes in Cyprus (*N* = 1117). Commonly, a smaller rural community is a single postcode while in urban areas, postcodes have an average population of around 1500 people and 530 households.

Finally, a variation of the MacArthur Scale of Subjective Social Status [31, 32] was used to assess the individual’s perception of relative standing of their neighbourhood in the social hierarchy. Commonly, such measures represent the social structure as a “ladder”, often with 10 steps, and responders are asked to locate themselves, or in this case their neighbourhood, on the ladder considering that the top represents those neighbourhoods who are best off (most privileged) and the bottom the neighbourhoods who are worst off (most disadvantaged). Such measures are considered particularly relevant as they represent an internalized perception of position in the social hierarchy through a cognitive “synthesis” of several factors not always easy to capture by other indicators and through a process of social comparison, in this case, with other neighbourhoods [33].

### Data collection process

The online questionnaire was posted on a Facebook page created specifically for this purpose (Cyprus Neighbourhoods and Health study, @CyNeighborhoodsHealth). To maximise reach, the study was promoted in three ways: (a) through a press-release to the mainstream media reporting the signing of Memorandum of Understanding between the University and the WHO Health Cities coordinating office of the Cyprus Ministry of Health, (b) with the use of paid Facebook ads and (c) forwarding to municipalities with the request to promote on their official websites and social media channels. Paid Facebook ads targeted both a wide audience (i.e. location “Cyprus” and age “over 18 years of age”) as well as more targeted audience using a range of pre-selected options under ‘Hobbies and Activities”, such as Charity and causes, Community issues, Environmentalism, Politics, Sustainability, Volunteering, etc.

### Ethical considerations

Participation in the online survey was voluntary and anonymous and did not require the responder to provide an email address or any other personal identification. The study was performed in accordance to the principles of the Declaration of Helsinki. Written information about the purpose of the study, data collection and analysis as well as information about how to withdraw participation at any stage were provided on the first page of the online questionnaire. Participants provided informed consent to continue by actively ticking a box to opt in. Since no personal identifiers were recorded, participants were encouraged to take a note of the “timestamp” (i.e. date and exact time of submission) should they wish to withdraw their participation even after submitting their responses. By continuing, the participants also declared they were completing the survey only once and agree for their assessment to be included in the analysis along with all other ratings. Participants could terminate their participation at any point by closing their browser, in which case no data were recorded. The particular study as well as the full protocol for the wider CyNOTes programme was approved by the Cyprus National Bioethics Committee (ΕΕΒΚ ΕΠ 2018.01.131).

### Statistical analysis

The socio-demographic profile of participants was compared to the expected distribution of these characteristics in the Cypriot population according to the 2011 census. Postcodes, as provided by participants, were checked for inconsistencies based on municipality/ community and district of residence and were classified as urban or rural based on administrative criteria and linked to a series of census-based indicators that were publicly available at postcode level at the Statistical Services of Cyprus. Specifically, these include indicators of the built environment (% of houses built prior to 1980, % of apartment blocks and mixed-used buildings) and socio-demographic composition with a focus on specific population groups often associated with socio-economic disadvantage (% of single-parent households and % non-Cypriot population and % of population aged 65 years and over).

Place Standard ratings were analysed both by calculating summary statistics (mean, SD, median, IQR, min, max) as well as frequencies of responses across the 7-point scale for each of the 14 items. A total score was also calculated as the sum of all 14 ratings (theoretical range: 14–98). Item-item and item-total correlations were examined by calculating Spearman’s correlation coefficient. Ratings of 1–2 (i.e. least satisfied), 3–5 (around midpoint) and 6–7 (most satisfied) were grouped for the purposes of further analysis. The extent to which the frequency of low ratings (1 or 2) is inversely associated with neighbourhood’s social position (as assessed by participants) was explored and the likelihood of responders to express dissatisfaction along the social disadvantage continuum was estimated in logistic regression models.

After assessing sampling adequacy with the Kaiser–Meyer–Olkin coefficient and the Barlett’s test of sphericity, Exploratory Factor Analysis (EFA) with principle component extraction and orthogonal rotation was performed to assess the dimensionality of the Place Standard. The criteria for the number of factors to retained were: eigenvalues greater than 1, examination of the scree plot and factor loadings of 0.4 or higher. The factors were interpreted in terms of capturing distinct aspects of the neighbourhood environment and factor scores were calculated as the unweighted sum of items with high loadings on each factor. The internal consistency of the overall scale and identified subscales were assessed using Cronbach’s alpha coefficient. K-means cluster analysis was also performed to classify responders’ neighbourhoods in relatively homogeneous groups based on all 14 Place Standard ratings. Different number of clusters were specified to identify clusters that are as distinct from each other as possible.

Differences in overall and sub-scale mean scores by (a) socio-demographic characteristics of the participants, (b) participants’ subjective assessment of neighbourhood social position and (c) across quartiles of neighbourhoods objectively classified according to census indicators were explored using one-way ANOVA. Linear regression was used to explore the association between mean scores and area-level characteristics before and after adjusting for individual socio-demographic characteristics in order to control for the confounding effect of the potentially differing profile of responders across these groups of neighbourhoods.

## Results

A total of 492 people (62% women, 37% men) responded to the online questionnaire, the vast majority (90.6%) between the ages 25–64 [mean (SD): 42.2 (12.4) years, median: 40, IQR: 33–55). Due to the design of the study, participation rate cannot be estimated, but according to Facebook statistics, the paid ads reached 8932 people across all age-groups with an equal proportion in terms of gender, resulting in 523 engagements over a period of two weeks. Missing socio-demographic information and item ratings were limited to 3–5 casewise and 7 listwise, leaving 485 complete questionnaires for analysis. Responses originated from 254 postcodes, representing 21.7% of all postcodes (*N* = 1117). With only a small number of wrong or inconsistent postcodes (*N* = 14), the remaining 240 (94.5%) were linked to area-level census indicators. While, the urban–rural population distribution in Cyprus is 67% vs 33%, as many as 85.6% of the ratings in this survey concerned an urban neighbourhood. There was higher participation from the city of Limassol (where the University is based) and Nicosia (capital city), while only 10% of responses originated from other districts.

Table 1 presents the socio-demographic characteristics of the participants along with the expected distribution of these variables according to the 2011 census, where available, for the total population as well as in people aged 24–64 which corresponds more closely to the age profile of the participants. More than half of the responders (52.4%) reported that they have been living in their current address for over 10 years. They were also more likely to be home owners (75.4%) and live in a detached or semi-detached house than an apartment (67.3%) which nevertheless is consistent with the census. With regard to household size, marital and occupational status, the profile of participants also appeared relatively consistent with the census. However, in terms of educational attainment, survey participants were much more likely to be University graduates (81%) compared to what would be expected for this age group.

**Table 1:** Socio-demographic characteristics of the participants (*N* = 492)

Variable	Variable Categories	N	%	2011 census†	2011 census – ages 25–64
**Gender**	**Male**	182	37.0%	48.6%	47.8%
	**Female**	307	62.4%	51.4%	52.2%
	**Not reported**	3	0.6%		
**Age**	** < 25**	16	3.3%	30.6%	
	**25–34**	130	26.4%	17.1%	30.5%
	**35–44**	166	33.7%	14.5%	25.8%
	**45–54**	76	15.4%	13.5%	24.0%
	**55–64**	74	15.0%	11.0%	19.7%
	**65 + **	26	5.3%	13.3%	
	**Not reported**	4	0.8%		
**Nationality**	**Cypriot**	447	90.9%	79.4%	68.7%
	**Not Cypriot**	42	8.5%	20.3%	30.5%
	**Not reported**	3	0.6%		
**Marital status**	**Married/Cohabiting**	336	68.3%	50.0%	71.5%
	**Single**	117	23.8%	41.0%	19.7%
	**Divorced/ Widowed**	36	7.3%	8.2%	7.8%
	**Not reported**	3	0.6%		
**Household size**	**Single-person**	67	13.6%	20.8%	17.9%
	**Two-person household**	120	24.4%	30.9%	22.9%
	**Three-person household**	121	24.6%	18.2%	20.1%
	**Four-person household**	131	26.6%	17.5%	22.5%
	** ≥ 5-person household**	48	9.8%	12.6%	16.5%
	**Not reported**	5	1.0%		
**Educational attainment**	**Up to secondary**	40	8.1%	67.6%	59.3%
	**Tertiary-College**	51	10.4%	10.3%	12.8%
	**Tertiary-University**	143	29.1%	14.6%	18.1%
	**Postgraduate degree**	208	42.3%	5.1%	6.9%
	**Doctoral degree**	47	9.6%	0.5%	0.7%
	**Not reported**	3	0.6%		
**Employment status**	**Full-time employment**	357	72.6%	52.6%	79.5%
	**Part-time employment**	55	11.2%		
	**Unemployment**	17	3.5%	6.5%	7.6%
	**Not active/ Retired**	60	12.2%	38.4%	18.9%
	**Not reported**	3	0.6%		
**Financial difficulties ¥**	**No**	312	63.4%		
	**Yes**	177	36.0%		
	**Not reported**	3	0.6%		
**House tenure ‡**	**Owner-occupied**	371	75.4%	67.8%	67.9%
	**Privately renting**	100	20.3%	19.5%	22.4%
	**Other**	18	3.7%	11.6%	8.4%
	**Not reported**	3	0.6%		
**House type ‡**	**Detached**	219	44.5%	40.1%	
	**Semi-detached**	112	22.8%	21.2%	
	**Block of ≤ 8 apartments**	85	17.3%	28.7%	
	**Block of > 8 apartments**	73	14.8%		
	**Not reported**	3	0.6%		
**Residence in current address¥**	**More than 10 years**	258	52.4%		
	**5–10 years**	92	18.8%		
	**3–5 years**	47	9.6%		
	**1–3 years**	61	12.4%		
	**Less than 1 year**	31	6.3%		
	**Not reported**	3	0.6%		
**District of residence**	**Nicosia**	216	43.9%	38.9%	39.6%
	**Limassol**	225	45.7%	28.0%	27.9%
	**Other**	51	10.4%	33.1%	32.5%
**Urban–Rural**	**Urban**	421	85.6%	67.4%	68.7%
	**Rural**	71	14.4%	32.6%	31.3%

^†^ Educational attainment and employment status refer to the population over 15 years of age, the rest of the variables refer to all age groups. ‡ Census estimates for house tenure and type are expressed as a proportion of total number of households and not population. Estimates for housing type by age-group of interest was not readily available in the 2011 census reported figures. ¥ Proportion of people reporting financial difficulties and length of residence in current address are not available from the 2011 Cypriot census

Table 2 presents summary statistics of the participants’ ratings across the 14 Place Standard items (with lower ratings denoting more room for improvement and thus more dissatisfaction with current state) as well as the total score (theoretical range: 14–98). Even though there was wide variability, ratings were generally low with averages close or consistently below the midpoint across all items. Sense of safety in the neighbourhood (Q12: *“Do I feel safe here?”* with reference to crime and anti-social behaviour among others) was the only item with an average rating above the midpoint (M = 4.35, SD = 1.74). All other domains were not rated as favourably with the lowest average scores recorded for “influence and sense of control” (Q14: *“Do I feel able to take part in decisions and help change things for the better?”*) with Mean score (SD) = 2.44 (1.59), followed by “public transport” (Q2: *“Does public transport meet my needs?”* referring to affordable, reliable and well-connected services), with Mean (SD) = 2.47 (1.50). The percentage of survey participants who thought that there is large room for improvement in these two aspects (i.e. a rating of 2 or lower) is as high as 60.3% and 58.0% respectively. Table 1 also shows the frequency distribution of responses across low (1–2) vs high (6–7) ratings. As many as 33%-51% of participants gave a low rating in a further seven domains. In contrast, only one in every ten participants rated their neighbourhood environment favourably in eleven out of 14 domains. Aspects rated relatively more favourably were “Safety”, “Natural space” and “Work and Local economy”, for which at least one in four people gave a comparatively high rating.

**Table 2:** Assessment of the 14 Place Standard single-item dimensions of the neighborhood environment on a scale of 1: large improvement to 7: little improvement (*N* = 488–491)

	**Summary statistics**				**Percentage reporting …**		
**Place Standard Tool items**	**N**	**Mean (SD)**	**Median**	**Min–Max**	**Large improvement (responses 1–2)**	**Around midpoint (3–5)**	**Little improvement (responses 6–7)**
**Q1: Moving around**	491	2.87 (1.82)	2	1–7	51.3%	37.9%	10.8%
**Q2: Public transport**	488	2.47 (1.50)	2	1–7	58.0%	38.1%	3.9%
**Q3: Traffic & Parking**	489	3.06 (1.72)	3	1–7	43.8%	47.0%	9.2%
**Q4: Streets & Spaces**	489	3.00 (1.68)	3	1–7	43.6%	48.3%	8.2%
**Q5: Natural space**	489	3.38 (1.98)	3	1–7	41.5%	38.7%	19.8%
**Q6: Play & Recreation**	491	3.18 (1.78)	3	1–7	41.3%	45.8%	12.8%
**Q7: Facilities & Amenities**	490	3.35 (1.70)	3	1–7	35.3%	52.9%	11.8%
**Q8: Work & Local economy**	490	3.75 (1.72)	4	1–7	25.9%	56.1%	18.0%
**Q9: Housing & Community**	490	3.50 (1.72)	4	1–7	33.3%	54.3%	12.4%
**Q10: Social contact**	491	3.14 (1.76)	3	1–7	43.8%	44.6%	11.6%
**Q11: Identity & Belonging**	491	3.43 (1.81)	3	1–7	35.2%	50.9%	13.8%
**Q12: Feeling safe**	491	4.35 (1.74)	5	1–7	17.9%	50.8%	31.1%
**Q13: Care & Maintenance**	491	3.21 (1.72)	3	1–7	38.9%	51.3%	9.8%
**Q14: Influence & Sense of control**	491	2.44 (1.59)	2	1–7	60.3%	34.0%	5.7%
**Total score (theoretical range: 14–98)**	**485**	**44.94 (15.09)**	**45**	**17–89**			
	**N**	**Mean (SD)**	**Median**	**Min–Max**	**Relatively disadvantaged (1–3)**	**Around midpoint (4–7)**	**Relatively privileged** **(8–10)**
**Subjective assessment on 1–10 ladder of neighborhood’s social position**	491	5.91 (2.18)	6	1–10	17.1%	58.1%	24.6%

With regard to the participants’ subjective assessment of neighbourhood’s social position (NSP), 24.6% placed their neighbourhood at steps 8 or 9 (i.e. relatively privileged), with no one choosing the top step. The majority (58.1%) placed their neighbourhood between steps 4–7, while 17.1% placed it at the bottom three steps (i.e. relatively disadvantaged compared to other neighbourhoods). Subjective assessment of neighbourhood’s social position was positively correlated with all Place Standard domains (*ρ* = 0.3–0.5) – see Table 3. The only exception for which a lower correlation was observed was with regard to Public Transport. This may not be surprising given the fact that the transport network and services in Cypriot cities and communities are neither well-developed nor widely used by the public, and, as such, may not be generally considered a defining feature of a neighbourhood’s social position. Neighbourhood’s social position (as assessed by participants) was more strongly correlated with the total Place Standard score (*ρ* = 0.6) rather than any single item.

**Table 3:** Spearman’s correlation coefficient between the 14 domain scores of the neighborhood’s environment and subjective assessment of neighborhood’s social position

Place Standard	Q1	Q2	Q3	Q4	Q5	Q6	Q7	Q8	Q9	Q10	Q11	Q12	Q13	Q14	Total score	Neighborhood social position
Q1: Moving around	1															
Q2: Public transport	0.27	1														
Q3: Traffic & Parking	0.37	0.24	1													
Q4: Streets & Spaces	0.39	0.27	**0.46**	1												
Q5: Natural space	**0.48**	0.24	0.33	**0.51**	1											
Q6: Play & Recreation	0.39	0.30	0.31	**0.47**	**0.69**	1										
Q7: Facilities & Amenities	0.21	0.27	0.33	**0.40**	**0.40**	**0.41**	1									
Q8: Work & Local economy	0.17	0.25	0.17	0.33	0.22	0.33	**0.40**	1								
Q9: Housing & Community	0.25	0.26	0.37	0.36	0.32	0.26	0.33	0.36	1							
Q10: Social contact	0.27	0.21	0.26	0.34	**0.40**	**0.43**	0.25	0.38	0.36	1						
Q11: Identity & Belonging	0.27	0.21	0.30	**0.46**	**0.40**	0.37	0.34	0.32	0.33	**0.60**	1					
Q12: Feeling safe	0.21	0.12	0.35	0.36	0.34	0.28	0.35	0.26	0.37	0.33	**0.42**	1				
Q13: Care & Maintenance	0.25	0.22	**0.42**	**0.52**	**0.42**	**0.40**	**0.42**	0.25	**0.49**	0.33	**0.42**	**0.51**	1			
Q14: Influence & Sense of control	0.25	0.18	0.31	**0.40**	**0.40**	**0.40**	0.34	0.20	0.35	0.37	0.37	0.29	**0.44**	1		
Total Place Standard score	**0.55**	**0.45**	**0.60**	**0.72**	**0.71**	**0.70**	**0.62**	**0.53**	**0.62**	**0.64**	**0.67**	**0.59**	**0.69**	**0.60**	1	
Neighborhood social position	0.37	0.17	0.36	**0.48**	0.39	0.38	0.37	0.36	0.30	0.24	**0.43**	**0.50**	**0.47**	0.27	**0.60**	1

Correlations ≥ 0.40 are indicated in bold to facilitate interpretation

Pairwise correlations between Place Standard items were positive and in the magnitude of 0.2–0.7. Inter-item and item-total correlations are also presented in Table 3. The strongest correlations were observed between conceptually related domains. For instance, “Natural space” (Q5: *“Can I regularly experience good-quality natural space?”*, which would include parks and other green spaces) is strongly correlated (*ρ* = 0.69) with “Play and Recreation” (Q6: “*Can I access a range of spaces with opportunities for play and recreation?”).* Similarly, “Social Contact” (Q10: “*Is there a range of spaces and opportunities to meet people?”*) is more strongly correlated with “Identity and Belonging” (Q11: *“Does this place have a positive identity and do I feel I belong?”*).

Cronbach’s alpha coefficient for internal consistency for the overall scale was 0.88. Kaiser–Meyer–Olkin coefficient for sampling adequacy was 0.885 and the Bartlett’s test of sphericity was statistically significant (*p*-value < 0.001), supporting appropriateness for factor analysis. Table 4 presents the factor structure of the Place Standard. The analysis revealed a clear and readily interpretable dimensionality of four factors with an eigenvalue greater than one. There was very little cross-loading and the four factors explained 61.6% of the total variance. Six items loaded on the first factor, explaining 19.5% of the variance. The first factor (6 items) mainly taps on aspects or features related to the Built Environment (Traffic & Parking, Streets & Spaces, Housing & Community) as well as their overall level of Care & Maintenance (item Q13 directly refers to whether *“buildings and spaces”* are well-cared for). Interestingly, neighbourhood safety also loaded on this factor. While the specific item refers to general “feelings of safety”, prompt questions provided to assist participants in thinking of this aspect of neighbourhood refer to “derelict property”, “safe routes”, which would also include, for example, well-lit paths frequented by people, and other design features of public and communal spaces (e.g. “spaces overlooked by buildings”). Furthermore, while “safety” relates to social activity, it should be noted that other potentially related items in the Place Standard refer to positive aspects of the social environment rather than disorder (e.g. anti-social and delinquent behaviour). The only item in the first factor whose link to the built environment appears conceptually weak is “Influence and Sense of control”. While the item loading was rather low (and close to the pre-set cut-off point of 0.4), neither extracting three or five factors revealed an improved structure or interpretation. In fact, extracting more factors results in this item loading on its own as a fifth factor.

**Table 4:** Four-factor dimensionality of the Place Standard Tool in the rotated component matrix

Place Standard	Factor 1: Built environment (6 items)	Factor 2: Physical environment (3 items)	Factor 3: Social environment (2 items)	Factor 4: Service environment (3 items)
Q1: Moving around		**0.73**		
Q2: Public transport				**0.74**
Q3: Traffic & Parking	**0.65**			
Q4: Streets & Spaces	**0.51**	*0.49*		
Q5: Natural space		**0.76**		
Q6: Play & Recreation		**0.71**		
Q7: Facilities & Amenities	*0.43*			**0.45**
Q8: Work & Local economy			*0.47*	**0.69**
Q9: Housing & Community	**0.61**			
Q10: Social contact			**0.78**	
Q11: Identity & Belonging			**0.69**	
Q12: Feeling safe	**0.69**			
Q13: Care & Maintenance	**0.76**			
Q14: Influence/Sense of control	**0.44**			
% variance explained (61.6%)	**19.5%**	**17.1%**	**14.0%**	**11.0%**
Cronbach’s alpha internal consistency (0.88)	**0.798**	**0.765**	**0.749**	**0.580**

Notes: ΚΜΟ Measure of Sampling Adequacy = 0.885; p-value Bartlett’s test for Sphericity < 0.001

Three items loaded on the second factor, explaining a further 17.1% of the variance, namely Natural space, Play & Recreation, and Moving around (which refers to walking and cycling). This was termed “Physical Environment”, as it captures parks, green areas and other spaces such as walking paths and cycling routes which, rather than a streetscaping feature in the context of a Cypriot city, they are more likely to be found within parks, designated nature areas or alongside dry river beds (linear parks). The two Place Standard items which tap on the “Social Environment”, namely Social contact and Identity & Belonging, loaded on the third factor, explaining a further 14% of the variance. Lastly, the remaining three items (Facilities & Amenities, Public Transport and Work & Local economy) loaded on the fourth factor, explaining a further 11% of the variance. Work and Local economy also loaded on the “Social Environment”. While interpretable, since a community with an active local economy with shops, restaurants and other meeting places may presumably provide additional opportunities for social contact, its loading (0.47) was much lower than the next higher loading on this factor (0.69), while it clearly had a higher loading on the “Service Environment” (0.69). All factors had high internal consistency with Cronbach’s alpha coefficient ranging from 0.6 (service environment) to 0.8 (built environment).

**Fig. 1:**
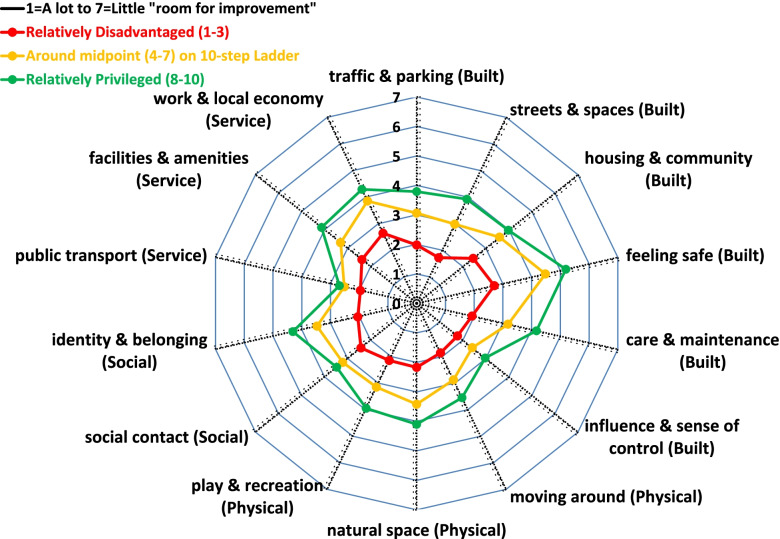
Radial plot of mean Place Standard ratings according to the participants’ subjective assessment of neighbourhood’s social position

**Fig. 2:**
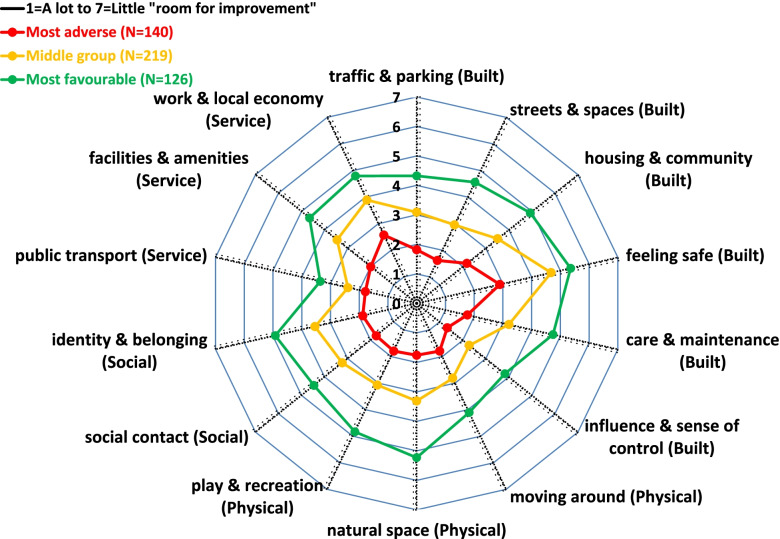
Radial plot of mean Place Standard ratings across three profile clusters of neighbourhoods

Survey participants who placed their neighbourhood on the three top steps (8–10) of the social position ladder consistently rated all aspects of the neighbourhood environment more favourably. Using the characteristic Place Standard radial plot, Fig. 1 presents the difference in mean ratings according to the participants’ subjective assessment of their neighbourhood’s social position (NSP). There was a clear stepwise pattern by NSP, with the lowest scores consistently recorded among participants who placed their neighbourhood on the bottom three steps of the ladder (1–3). With the exception of Public Transport, all observed differences were statistically significant at the 1% level. The largest differences were observed in terms of “Safety” and “Street & Spaces”, while the smallest were observed in terms of “Social contact”. K-means cluster analysis identified three groups of neighbourhoods based on the participants’ ratings. Figure 2 presents the Place Standard profile of these clusters. Interestingly, differences were clearly apparent in all aspects and no particular feature was more defining of the clusters than any other. Each of these neighbourhood profiles was related with up to two steps lower on the 1–10 subjective social position ladder. Specifically, the Mean (SD) of the NSP across neighbourhoods from most (*N* = 126) to least favourable ratings (*N* = 140) were 7.39 (1.42), 6.19 (1.63) and 4.12 (2.22) respectively; *p*-value < 0.001.

The magnitude of differences by subjective NSP was further explored in terms of the percentage of survey participants who reported that there is large room for improvement in a certain aspect or feature (i.e. rated 2 or lower on the 1–7 Place Standard scale). A clear stepwise association between NSP and all aspects of place was observed with increasing likelihood of expressing dissatisfaction with neighbourhood along the social position continuum. Table S1 (see Additional file 2) presents the frequency of low ratings for each neighbourhood aspect by neighbourhood’s social position and the respective odd ratios, as estimated in logistic regression models. In fact, this consistent stepwise pattern largely holds even across every step of the social position ladder (see Table S2, Additional file 2) and appears both in the case of features rated more favourably (e.g. safety and social contact) as well as generally less favourably (e.g. influence and sense of control). For instance, only 6.3% of the participants who placed their neighbourhood at the top of the ladder expressed concern about safety, whereas this figure rises incrementally along the social position continuum with 1.7 times (95% CI 1.5–1.9) more likely to rate neighbourhood safety lower at every preceding step of the ladder, to a high of 85% among those who placed their neighbourhood at the bottom. Similarly, even though a generally higher percentage of participants rated “influence and social control” low, including those at the top of the social position ladder (48.8% in steps 8–10), the equivalent figure among those at the bottom (steps 1–3) is 81.0%, representing a statistically significant difference in the odds ratio scale of 4.5 times (95% CI 2.3, 8.6) more likely to express dissatisfaction about sense of participation at the lower end of the social position scale.

Table 5 presents mean differences in the four factor scores and overall Place Standard score by subjective NSP. To facilitate comparisons, scores were transformed to a 0–100 scale due to the different number of items in each sub-scale. Average scores were generally low (45–50) even among those who, according to their own assessment, lived in relatively privileged neighbourhoods. Lower average scores were recorded by descending social position across all domains (built, physical, social and service environment). Among those who placed their neighbourhoods at the bottom of the ladder (1–3), average scores were in the range of 17–22. All observed differences were statistically significant (p value for linear trend < 0.001) with average scores incrementally lower by around 4 (service environment) to 7 (built environment) points per every step on the 1–10 ladder.

**Table 5:** Observed differences in neighborhood environment scores (total scale and sub-scales on a 0–100 scale) by subjective assessment of neighborhood’s social position

Subjective assessment of neighborhood’s social position (1–10 step ladder)	Built environment (6 items)	Physical environment (3 items)	Social environment (2 items)	Service environment (3 items)	Total Score (projected on 1–100)
	**Mean (SD)**	**Mean (SD)**	**Mean (SD)**	**Mean (SD)**	**Mean (SD)**
**Relatively disadvantaged (1–3)**	17.9 (16.3)	17.2 (21.0)	20.5 (24.6)	22.0 (18.1)	18.9 (15.3)
**Around midpoint (4–7)**	37.9 (16.2)	35.8 (23.4)	39.0 (24.8)	37.0 (18.1)	37.2 (14.6)
**Relatively privileged (8–10)**	50.0 (19.6)	47.8 (26.0)	48.2 (26.4)	45.0 (20.6)	48.2 (17.0)
***p*-value**	< 0.001	< 0.001	< 0.001	< 0.001	< 0.001
	**Coefficient** **(95% CI)**	**Coefficient** **(95% CI)**	**Coefficient** **(95% CI)**	**Coefficient** **(95% CI)**	**Coefficient** **(95% CI)**
**Per category increase (95% CI)**	15.6 (13.3, 18.0)	14.9 (11.6, 18.2)	13.3 (9.9, 16.8)	11.2 (8.5, 13.8)	14.2 (12.1, 16.4)
***p*-value for trend**	< 0.001	< 0.001	< 0.001	< 0.001	< 0.001
**Per unit increase across 1–10 ladder**	6.7 (4.5, 5.9)	5.4 (4.5, 6.4)	4.6 (3.6, 5.6)	3.9 (3.1, 4.6)	4.9 (4.3, 5.5))
***p*-value for trend**	< 0.001	< 0.001	< 0.001	< 0.001	< 0.001

While no clear pattern in the ratings was observed according to the gender or age of the participants, certain socio-demographic characteristics appeared to be more consistently associated with neighbourhood ratings. Observed differences in ratings according to participants’ characteristics are presented in Table S3 (see Additional file 3). Home owners and people with higher educational attainment tended to rate several aspects of the neighbourhood environment more favourably. Furthermore, there was a consistent pattern of lower ratings in all domains among people who reported financial difficulties, with the exception of social environment. In fact, social environment scores were not associated with any socio-demographic characteristics.

Table 6 presents differences in mean ratings across quartiles of neighbourhoods objectively classified according to a series of census indicators. Differences are presented before and after adjusting for individual-level socio-demographic characteristics to control for the potential confounding effect of the differing profile of responders across these groups of neighbourhoods. The domain more consistently associated with all census indicators was the built environment, displaying a gradient of lower average scores across quartiles of neighbourhoods with increasing proportion of apartment blocks or mixed used buildings and a higher proportion of houses constructed pre-1980. Similarly, there was evidence of a social gradient in terms of specific population groups often associated with socio-economic disadvantage. Lower built environment scores were associated with increasing proportion of people aged 65 or over, single-parent households and non-Cypriot population in an area. The observed differences were not large in magnitude (i.e. commonly 10–12 points difference between lowest and highest quartile) but the pattern appeared consistent and, with the exception of older population, the associations persisted after adjusting for socio-demographic characteristics. Interestingly, both physical and service environment appeared to be positively associated with the proportion of apartment blocks/mixed used buildings; however, this may reflect the fact that, in Cypriot cities, walking paths, cycling routes, parks and other green areas tend to be located in more centrally-located areas which also have a much more active local economy compared to residential areas in the periphery. With regard to the social environment, other than higher ratings in rural vs urban areas, no associations were observed with any of the census indicators.

**Table 6:** Neighborhood environment scores by census indicators before and after adjusting for socio-demographic characteristics of the participants

Census variables	Classification of postcodes according to quartile levels of census indicators	Built environment score (6 items)	Physical environment score (3 items)	Social environment score (2 items)	Service environment score (3 items)	Total Place Standard score (14 items)
**Area-level variables before and after adjusting for individual-level variables† ‡**		**Mean (SD)**	**Mean (SD)**	**Mean (SD)**	**Mean (SD)**	**Mean (SD)**
**% pre-1980 construction**	**Q1 – Lowest**	41.3 (18.6)				38.5 (17.4)
	**Q2**	39.0 (19.1)				37.3 (16.6)
	**Q3**	36.0 (19.7)				37.2 (18.9)
	**Q4—Highest**	32.6 (20.4)				34.4 (18.7)
	***p*-value**	0.007				0.34
	**Unadjusted – Difference per quartile (95% CI); *p*-value**	-2.6 (-4.2, -1.0); *p* = 0.001				-1.2 (-2.7, 0.2); *p* = 0.09
	**Adjusted—Difference per quartile (95% CI); *p*-value**	-1.8 (-3.4, -0.2); *p* = 0.03				-1.1 (-2.6, 0.3); *p* = 0.12
**% apartment blocks-mixed used**	**Q1 – Lowest**	43.7 (22.9)	34.6 (25.5)		32.2 (21.4)	
	**Q2**	35.8 (19.5)	29.8 (24.1)		36.0 (19.4)	
	**Q3**	38.0 (18.2)	36.3 (24.6)		38.6 (19.5)	
	**Q4—Highest**	31.7 (15.5)	40.3 (25.7)		38.3 (18.8)	
	***p*-value**	< 0.001	0.02		0.06	
	**Unadjusted – Difference per quartile (95% CI); *p*-value**	-3.2 (-4.8, -1.6); *p* < 0.001	2.4 (0.4, 4.5); *p* = 0.02		2.0 (0.4, 3.6); *p* = 0.02	
	**Adjusted—Difference per quartile (95% CI); p-value**	-2.9 (-4.6, -1.2); *p* < 0.001	2.2 (0.2, 4.2) *p* = 0.03		2.0 (0.4, 3.6); *p* = 0.01	
**% single-parent households**	**Q1 – Lowest**	40.6 (20.1)	39.4 (26.9)			38.9 (17.9)
	**Q2**	41.2 (19.4)	33.4 (22.9)			38.6 (16.6)
	**Q3**	34.2 (19.2)	36.9 (27.4)			35.1 (18.8)
	**Q4—Highest**	32.9 (18.8)	32.2 (24.2)			33.9 (16.9)
	***p*-value**	0.001	0.11			0.08
	**Unadjusted – Difference per quartile (95% CI); *p*-value**	-2.7 (-4.3, -1.1); *p* = 0.001	-1.8 (-3.9, 0.2); *p* = 0.08			-1.8 (-3.2, -0.3); *p* = 0.02
	**Adjusted—Difference per quartile (95% CI); p-value**	-2.3 (-3.8, -0.7); *p* = 0.005	-1.9 (-4.0, -0.1); *p* = 0.06			-1.7 (-3.1, -0.2); *p* = 0.02
**% non-Cypriot population**	**Q1 – Lowest**	40.2 (19.7)	31.4 (23.2)			
	**Q2**	40.1 (20.0)	36.5 (27.2)			
	**Q3**	36.3 (21.4)	35.0 (24.8)			
	**Q4—Highest**	33.7 (17.7)	39.1 (26.4)			
	***p*-value**	0.03	0.13			
	**Unadjusted – Difference per quartile (95% CI); *p*-value**	-2.3 (-3.9, -0.7); *p* = 0.005	2.2 (0.1, 4.2); *p* = 0.04			
	**Adjusted—Difference per quartile (95% CI); p-value**	-1.7 (-3.3, -0.0); *p* = 0.05	2.0 (-0.1, 4.0); *p* = 0.06			
**% population aged 65 or over**	**Q1 – Lowest**	41.7 (18.4)				
	**Q2**	36.5 (19.0)				
	**Q3**	33.8 (21.1)				
	**Q4—Highest**	36.9 (19.4)				
	***p*-value**	0.02				
	**Unadjusted – Difference per quartile (95% CI); *p*-value**	-1.7 (-3.3, -0.1); *p* = 0.04				
	**Adjusted—Difference per quartile (95% CI); p-value**	-0.81 (-2.4, 0.8); *p* = 0.32				
**Urban–rural place of residence**	**Urban (*Ν* = 379)**	35.8 (19.8)		36.3 (26.2)	37.1 (19.5)	
	**Rural (*Ν* = 70)**	45.1 (22.7)		43.5 (27.6)	30.8 (21.4)	
	***p*-value**	< 0.001		0.04	0.02	
	**Unadjusted – Difference per quartile (95% CI); *p*-value**	-8.7 (-13.7, -3.7); *p* = 0.001		-6.3 (-13.1, -0.4); *p* = 0.07	6.6 (1.5, 11.6); *p* = 0.01	
	**Adjusted—Difference per quartile (95% CI); p-value**	-7.6 (-12.6, 2.7); *p* = 0.003		-6.4 (-13.1, 0.4); *p* = 0.06	6.9 (0.3, 7.8); *p* = 0.007	

## Discussion

### Main findings

With the exception of “safety”, participants from 254 different postcodes (21.7% islandwide) did not rate other features of the residential environment favourably, with lowest scores for “public transport” and “influence and sense of control”. A clear dimensionality of Built, Physical, Social and Service environment supports the construct validity of the Place Standard. People who placed their neighbourhood lower on the social position ladder were consistently more likely to rate their neighbourhood less favourably across all items and domains. Subjective neighbourhood social position was more strongly correlated with the overall Place Standard score rather than any single item, which may suggest that participants intuitively take several aspects into consideration in their relative assessment. A social gradient, partly supporting the criterion validity of the tool, was also evident according to census-based built (e.g. apartment blocks, mixed used buildings, pre-1980 housing) or socio-demographic area characteristics (e.g. single-parent households) but appeared more consistent in terms of features of the built rather than physical, social or service environment.

### Dimensionality of the Place Standard

The Place Standard has been used across different settings in at least 14 European countries, more commonly in the context of community engagement and development. With some exceptions, aspects of the tool’s metric properties are not reported in the published literature. For instance, in the online citizens’ perception survey in Skopje, North Macedonia, the internal consistency of the overall scale was reported (Cronbach’s alpha coefficient for internal consistency = 0.892), which is similar to the figure reported here. In our study, moderate to high correlations were also observed among conceptually related PST items, indicating good convergent validity. Our study also showed a readily identifiable and interpretable dimensionality of four sub-scales, all with high internal consistency. Other than supporting the scale’s construct validity, the achieved variable reduction (from 14 district ratings to 4 theoretical constructs) may simplify and facilitate the further use of the scale in the context of research studies.

### Comparison of the Place Standard with other neighbourhood quality metric tools

There are several neighbourhood tools in the literature used to survey residents’ perceptions about the quality of the residential environment or, conversely, problems with negatively phased items, such as the Neighbourhood Problems Scale [34]. Some of these tools have been purposefully developed through a psychometric and ecometric validation process, such as the Neighbourhood Scale by the Observatory for Urban Health, Belo Horizonte, Brazil [10], whereas in other cases a small number of items pertaining to specific features of interest are selected as best fit for the purposes of the particular study [8, 9, 34, 35]. A particular nice element in the Place Standard, attributed to its roots in community development rather than metrics, which is rarely reflected in other neighbourhood environment scales, is the explicit reference to the aspect of equity with supplementary questions prompting the rater to consider the inclusiveness of Place (e.g. “…whatever their age, mobility, disability, sex, ethnic group, religious belief or sexuality”).

Other than feature-specific, findings from other neighbourhood studies are setting-specific, thus not allowing direct comparisons. Nevertheless, some comparison can be made in terms of the domains which are commonly assessed. For instance, in the study by Friche et al. (2013), a questionnaire with ten domains and 70 items (out of 84 items considered) was developed [10]. Even though there isn’t a direct match between the 14 one-item dimensions assessed in the Place Standard and the ten multi-item domains of the Neighbourhood scale (numbered below), a detailed examination reveals significant overlap, especially if the prompt questions included to assist in the rating of the core item of the Place Standard Tool (PST) are considered. For example, 1. Safety (2 items), 2. Violence (6 items) and 3. Social disorder (6 items) are jointly tapping on Place Standard’s “feeling safe”, 4. Walking environment (7 items) on PST’s “moving around”, 5. Social cohesion (6 items) on “social contact” and partly on “identity and belonging”, 6. Neighbourhood participation (11 items) jointly on “identity and belonging” and “influence and sense of control”, 7. Aesthetic quality and 8. Physical disorder tap jointly on PST’s “Care and Maintenance”, while 9. Quality of services (8 items) and 10. Neighbourhood problems jointly tap on, but do not cover completely, PST’s “public transport”, “facilities and amenities”, “work and local economy”, “traffic and parking”, “streets and spaces” and “play & recreation”. In contrast, PST’s “natural space” and “housing and community” do not seem to have a strong representation in the Neighbourhood Scale.

It should be noted that several of these domains are inter-related. For instance, feelings of safety and aesthetic quality would both impact on the walkability of a neighbourhood beyond aspects of the built environment (e.g. quality of pavements). In the case of the Neighbourhood Scale [10], this is reflected in an overlap in a number of items in the sub-scales, whereas in the case of the Place Standard, this complexity is reflected in the prompt questions which tap on related aspects (e.g. “*Do routes feel safe to use all year round and at different times of the day?*” tapping on the aspect of safety in the context of “Moving around”). Even focusing on physical activity alone, a review of qualitative studies identified an inter-play of influences across aspects of the built, physical, social and service neighbourhood environment [36]. This complexity is not always reflected in measurement scales and often the extent of this overlap may result in constructs that, despite having good internal consistency, might represent several different dimensions of Place. For example, the construct termed, “neighbourhood aesthetic quality” in Mujahid et al. (2007) consists of 6 items which are not exclusively about aesthetics and pertain to several different dimensions of the Place Standard, including “Care and Maintenance” (e.g. item “the building and homes are well-maintained) and “Play and Recreation” (e.g. item “there are interesting things to do in my neighbourhood”) [12].

### Residents’ perceptions of the residential environment

Previous studies have explored the extent to which citizens’ perceptions of their residential environment reflect objectively measured metrics using neighbourhood audits with mixed findings, both across studies [9, 11], as well as within studies in terms of different neighbourhood environment domains [9]. A systematic review on this issue, focusing only on physical activity outcomes, found generally low to moderate agreement between objective and perceived neighbourhood environment measures across 85 studies, concluding that these are not inter-changeable and may represent different constructs [37]. In fact, a recent study that designed a new questionnaire on active mobility, based on existing questionnaires and a typology of factors developed from the interviews with citizens, identified discrepancies whereby even standard items commonly used in walkability questionnaires, such as community life or even quality of sidewalks, were not strongly reflected in people’s perceptions [38].

While studies identified several socio-demographic as well as environmental factors that may relate to this disagreement, reported associations were not always consistent across studies. Other than highlighting that both approaches are necessary and complementary, the review concluded that it is important to explore the way different environments may influence perceptions and the extent to which these may be differential across socio-demographic groups [37]. While this aspect was beyond the scope of this study, one of the aims of the wider CyNOTes project is to explore residents’ perceptions across a stratified sample of audited Limassol neighbourhoods, selected along the socio-economic disadvantage continuum, as well as the association of audit and perception scores with health-related quality of life. It is unclear whether perceptions of place mediate or moderate the association between neighbourhood environment and health, but it is likely that both processes are at play [39, 40].

As part of the SPOTLIGHT project, virtual audits using Google Street View of 60 neighbourhoods across 5 European countries were compared to self-reported responses of around 6000 people across these neighbourhoods [9]. Focusing on 10 obesogenic features in the neighbourhood environment, the study found higher agreement in the case of the service environment (e.g. presence of food outlets, recreational facilities and other destinations), while there was greater mismatch in features with a higher degree of subjectivity pertaining to the built or physical environment (e.g. condition of pavements, litter and graffiti), with residents, for example, perceiving a highly walkable neighbourhoods by objective criteria as less walkable or vice versa. Noting this discordance, the authors highlight the importance of factoring residents’ perceptions beyond objective measures in community assessments since it is ultimately perceptions and their determinants that are important, which may include both socio-demographic and psychosocial factors. In this study, people who reported financial problems were consistently more likely to rate their neighbourhoods unfavourably. It is likely that this reflects actual disadvantaged neighbourhood conditions, also supported by the lower scores recorded in areas with higher socio-economic disadvantage as indicated by census indicators. However, previous studies have also identified that perceptions can be affected by personal circumstances. Kamphuis et al. (2010) [8] showed that across 14 neighbourhoods in Eindhoven, Netherlands, objective (audited) neighbourhood conditions largely explain the perceptions of unattractiveness and unsafeness among lower income groups, however mental well-being and psychosocial factors, including perceptions of neighbourhood social cohesion, also contributed. A qualitative study of the lived experience of 28 adults across five diverse neighbourhoods in Brussels developed a socioecological conceptual framework of how citizens experience and perceive their residential environment [41]. The study delineated complex and bi-directional interactions between neighbourhood aspects and mental well-being that involve an inter-play of both physical and social contextual aspects of place alongside institutional (e.g. role, responsibility and responsiveness on behalf of local authorities) and individual factors, including personal life and socio-economic circumstances, highlighting even more the need for participatory approaches both in gaining an in-depth understanding of citizens’ experience of engagement or disengagement as well as a process in itself in cultivating a sense of community.

Poortinga et al. (2017) found that residents’ attachment to neighbourhood was predicted by the overall quality of neighbourhood environment (audited using the REAT 2.0 tool) even though, interestingly, it was property-level (related to people’s personal space such as condition of houses and front yards) than street-level (related to public spaces) indicators that were more predictive [11]. Besides identifying the potentially important distinction between the public vs private realm of “place” and what people actually perceive as their “neighbourhood”, this also raises questions as to the determinants of attachment, as a pre-perquisite of taking action. In this study, the Place Standard’s “Identity and sense of Belonging”, which is the item that would best tap on attachment, showed positive but weak associations with other PST items, with the exception of “social contact”. Furthermore, while the ‘social environment’ construct (these two items) was inversely associated with the subjective assessment of neighbourhood’s social position, the observed variability in the scores did not appear to be associated with any individual- or area-level variables. Neighbourhood social cohesion and attachment are much more complex concepts with important nuances that cannot be adequately captured by one item. Hes et al. (2021) makes a distinction between “sense of belonging” which reflects the relationship between one’s self with place versus “place attachment” which reflects the collective relationship of the community with place, and thus its identity [17].

### Citizens’ participation

“*Influence and sense of control*” was the item that was rated lowest among the participants. While in recent years, several municipalities and communities across Cyprus have re-visited their approaches and have started to introduce community-led neighbourhood groups, it is fair to say that “community engagement”, more often than not, takes the ‘traditional’ form of public presentation of the local authorities’ planning with limited input or feedback from citizens, even though these sessions are commonly termed public consultations due to their open-call nature. Commonly, interested or concerned citizens who attend these public events are informed about the authorities plans and course of action, with little room for negotiation and without prior consultation with the wider community. With reference to Arnstein's (1969) ladder of participation [42], these activities, despite their seemingly participatory nature, represent ‘tokens’ and are perceived by the community as such. In fact, participation varies substantially and is largely dependent on the matter at hand and is not free from vested financial or political interests. While, concerns and/or suggestions may be discussed or sometimes even negotiated, especially in the face of strong opposition, there is no clear feedback channel between the community and the authorities and the process is not formally embedded in the decision-making process.

Nursey-Bray (2020) gives a detailed account of the principles, processes, tools and skills needed for community engagement [43], which is best understood as a continuum according to the International Association for Public Participation (www.iap2.org) that ultimately depends on the level of control in decision-making and ranges from providing information to the public about a problem and potential solutions at one end through consultation, involvement, collaboration and empowerment at the other end. Given that the PCT’s main purpose is to be used as a community engagement and “place-making” advocacy tool, it offers an opportunity to re-think, test and re-structure previously ineffective processes in Cyprus. It is important to mention that, in recent years, a number of neighbourhood projects have been established in Cyprus; however, these are either largely dependent on limited and competitive funding opportunities and/or are not always free from political motivations or aspirations. One example is the *“yiatilemeso.com”* [Greek: For Limassol] initiative, inspired and led by a local architect, which provides opportunities for citizens to participate in public consultations both in the context of organized physical events and debates as well as through an online discourse platform. Another example of a grassroot initiative is “*MY Square*” project, funded by the European Solidarity Corps through the Youth Board of Cyprus. This is a participatory action project bringing together the residents of the Mesa Yeitonia municipality (Limassol) through all stages of re-designing one of the municipality’s main squares. While discussing top-down and bottom-up approaches, Horgan and Dimitrijević (2020) reflect on whether any activity *“can be ultimately tokenistic if it is incompatible with the will of government and unable to influence real decision-making”* [44]. Thus, while tools, such as the Place Standard, are valuable in providing a common framework to structure discussions about place and health with communities, the extent of which this represents a “voice” or a “token” depends on the ability to truly embed the process in decision-making processes.

Due to the inherent volunteer bias in our study, and in the absence of relevant research evidence on this issue from Cyprus, no direct inference can be drawn as to whether the low sense of influence and control reflects the widespread perception of citizens’ in Cyprus. It should be noted, however, that this finding appears consistent with the only other comprehensive implementation of the Place Standard Tool in Cyprus we are aware of. Specifically, the Action Plan for the historic centre of Nicosia developed by the Cyprus Energy Agency as part of the Sustainable Development of Historic Areas (SUSHI) project of the European Climate-KIC action [45]. Influence and sense of control was also rated particularly low among the interviewed sample of 221 people who either live, work or visit the area, and second lowest to Natural spaces, perhaps not surprising given the inner-city profile of the surveyed area. Also, consistent with our findings, aspects of the built environment were also rated generally lower by the participants in the SUSHI study while, similarly to our findings, the social environment as well as sense of safety were the items rated comparatively higher compared to all the rest. Interestingly, according to Eurostat surveys, perceptions of safety among the Cypriot public (with reference to crime, violence and vandalism in the place of residence) have remained constant during the last decade (2010–2019), even though Cyprus is among a small number of EU countries that have seen increases in recorded homicides according to population size during the same period.

While drawing direct comparisons across different settings should be avoided, it is also worth mentioning that in the online Place Standard survey in Skopje, North Macedonia, the citizen participation aspect was also rated particularly low relative to other dimensions and second only to Traffic & Parking [25]. Specifically, in their study, Gjorgjev et al. (2020) reported that as many as 55.4% of the 278 participants identified the need for large improvements in this aspect (i.e. score of 1 or 2 on the 7-point scale) [25], a percentage comparable to the 60.3% of participants recorded in this study. Interestingly, the Skopje study juxtaposed the online Place Standard survey ratings with the ratings obtained during focus groups with citizens and municipality officials. Perhaps not surprisingly, the authors reported generally higher average scores across nearly all dimensions among those participating in focus groups compared to the online survey, including influence and sense of control. While it is not clear from the study’s report whether the higher level of satisfaction among focus group participants can be attributed to the actual process of participation itself, the authors concluded that the use of the Place Standard “increased knowledge and confidence among citizens and enthusiasm for active involvement in decision making”.

Recognising the importance of citizens’ participation, Horgan and Dimitrijević (2019), identify the need for “social innovation” [27], defined as, to quote, “*new solutions…that simultaneously meet a social need more effectively than existing solutions and lead to new or improved capabilities and relationships and better use of assets and resources*” [46] to support collaborative and inclusive approaches in urban planning and place-making. They discussed and juxtaposed the use of Place Standard along other place-based frameworks, focusing on the implications of different approaches involving conventional (e.g. face-to-face use of Place Standard with citizens and stakeholder groups) versus digital tools (such as Moscow’s Smart City “Active Citizen” platform) for community engagement for urban planning. In a case study, the authors assessed the citizens’ experience with the “Active Citizen” platform, an information-led mechanism that enables citizens’ participation in decision-making. While acknowledging the potential of such digital technologies, they identified issues around ownership, governance and participation, concluding that, to quote: “*While technology provides cheap and effective ways to engage citizens around issues that have little material impact on their day to day lives and future resilience, when decision-making is required on large issues such as renovation or displacement, there is no substitute for offline face-to-face engagement in a real-world setting*” [27]. This statement is further reinforced by the principles of “place-making”, which are less about passive and more about active participation in a collaborative process of co-creating shared values, perceptions, memories and traditions that give meaning to and connect people to geographic space [24]. Even in settings with much longer history and stronger tradition of place-based initiatives, community empowerment requires great attention to both breadth as well as depth of participation [47].

The Place Standard Process Evaluation report [18] also presents a series of five case studies from the first year of the PST use in Scotland covering a variety of contexts and scales to facilitate community engagement and/or capacity building with stakeholders (e.g. from small-scale planning with reference to a town centre or housing regeneration project to wider strategic city planning decision-making). A variety of delivery methods are described in these select case studies, either alone or in combination, i.e. open-door community focus groups, one-to-one walk-about consultations with citizens, invited stakeholder workshops and/or wider online surveys. In all cases, encouraging citizen participation and ensuring inclusive and representative reach across harder-to-reach groups were identified among the key challenges in successful implementation. However, another challenge identified was the active support by the senior management and various stakeholder groups. Since its launch, the Place Standard has been embedded in local planning practices by several Local Authorities in Scotland and this may have contributed in broadening the practitioners’ understanding of links between place and health. However, as suggested by a recent study [48], this enhanced awareness may have not been effectively translated into formal Strategic Environmental Assessment practices in the context of spatial planning, which may still remain narrowly focused on environmental risks rather than driven by a more holistic view of place effects on health.

An online survey has the advantage of reaching a larger number of people, and in the case of this study, there was participation from all over the island, even though the online survey was actively promoted for only two weeks. While this may be suggestive of the potential for scaling-up in the context of a needs assessment exercise, it is highly unlikely that online surveys can promote a sense of participation, especially as a stand-alone activity as in our study, compared to a structured and iterative process driven by a coalition of academic institutions, local authorities and advocacy groups in the context of wider community engagement and development. Large scale canvassing exercises may prove useful to survey perceptions and opinions to guide initial stages of planning, but they are no replacement for deeper engagement with communities. Furthermore, while they hold the potential to widen the reach, in terms of absolute participation, the extent to which this results in a more representative reach is unclear. Unlike the North Macedonia study [25] or several of the case studies from Scotland [18], which employed different formats of delivery in parallel, the Place Standard was delivered only in an online format in our study. Future efforts should concentrate on comparing different methods of community engagement and delivery of the PST (e.g. online remote format vs face-to-face group sessions or walk-abouts) with a particular focus on scale (e.g. city-wide vs specific neighbourhoods) and the implications on participation, engagement and inclusiveness. Furthermore, future studies should widen the research questions beyond community profiling to provide an in-depth understanding of the lived experience of residents by exploring social inequity both across as well as within communities.

### Strength and limitations

This is the first study to profile the residential environment in Cyprus and depict residents’ perceptions across several dimensions of place important for health and well-being. Due to the voluntary participation, selection bias cannot be ruled out; hence, moderate to low ratings across all dimensions may reflect the fact that the study provided an opportunity for people to register their complaints about neighbourhood problems. Even though the sample is not representative, there was participation from as many as one in five postcodes across the island from diverse neighbourhoods in terms of socio-demographic profile and social position according to participants’ own assessment. Since the study aimed to explore the dimensionality of the scale, it was deemed more important to gather responses from a larger and heterogeneous set of neighbourhoods. In the majority of cases, there was only one or two responses per postcode. As three or more participations were restricted only in the case of 50 postcodes, mixed-models with random effects to control for potential clustering at the neighbourhood level could not be used. Gathering opinions from a larger sample of residents from a smaller set of neighbourhoods in future studies would allow to explore the aggregated perceptions of people rating the same neighbourhood and, hence, ecometric properties of the tool including the intra-neighbourhood agreement [9, 10, 12].

A strength of the study is that census indicators were used to explore the extent to which neighbourhood ratings vary according to the built or socio-demographic characteristics of the area. However, limited by the lack of generally-accepted indices of social disadvantage in Cyprus, only a small set of indicators were considered, restricted by the public availability of data. Furthermore, it should be noted that people’s definition of neighbourhood differs from the administrative definitions used here. While postcodes are the smallest geographical unit for which census data are available, they are still likely to be larger than what people perceive as their neighbourhood. Despite that, the study has documented a social gradient in neighbourhood environment both in terms of people’s perceived social position of their neighbourhood compared to others as well as against a set of objective indicators. Neighbourhoods were assessed progressively worse at every lower step of the neighbourhood social position ladder. In fact, a stepwise, if not near linear, pattern was observed along the full length of the social position continuum for all Place Standard items to a larger or lesser degree. Even though common method-source bias cannot be excluded since the assessment of neighbourhood social position was also self-reported, this may also suggest that, through a process of social comparison, people consider all aspects of their neighbourhood environment holistically, further supported by the stronger correlation observed between subjective social position and the overall Place Standard score than any single item.

Unfortunately, in our study, we did not have the opportunity due to the remote format of delivery to gather any feedback on the actual usefulness of the Place Standard. Future work should also consider using the Place Standard in the context of participatory learning and action research with diverse communities and community groups.

## Conclusions

The Place Standard showed good metric properties in its first application to assess the quality of neighbourhood environment in Cyprus, with an interpretable dimensionality of features pertaining to the built, physical, social and service environment. Furthermore, a social gradient was evident according to both subjective (i.e. perceived social position of their neighbourhood) and objective indicators of socio-economic disadvantage (i.e. area-level census-based indicators). While this is suggestive of the inequity in the residential environment, the extent and magnitude of needs to be explored further employing a wider methodological toolbox which includes neighbourhood audits, qualitative and mixed-methods participatory research studies across socio-economically diverse neighbourhoods.

The Place Standard Tool may therefore be appropriate to use as measurement tool both in the context of Place profiling and its association with health as well as in the context of neighbourhood policy or programme initiatives along the full cycle from designing to evaluating their impact. In this first application, obtaining a sample from a heterogeneous sample of neighbourhoods was important for the purposes of ensuring variability in the measurement. However, future studies should focus on specific communities and ensure wider participation of citizens, with a focus on equity and inclusion of seldom-heard groups. Furthermore, in this internet-based study, analysis was based only on the quantitative ratings. Given the attention discussions about “place” gets in local politics and among community advocacy groups compared to the low attention it receives in the context of local public health research and practice, future applications of the Place Standard in Cyprus should consider testing and evaluating participatory action approaches with intersectoral representation involving local authority officials, inter-disciplinary community professionals and, of course, citizens of all ages and backgrounds.

## Supplementary Information

**Additional file 1.**

**Additional file 2.**

**Additional file 3.**

## Data Availability

The datasets used and/or analysed during the current study as well as the material, including the online (abridged) and booklet (full) version of the Greek edition of the Place Standard Tool are available from the corresponding author upon reasonable request.
