# Supplementary material for: Profiling the variability and inequity in the residential environment in Cyprus according to citizens’ ratings: a cross-sectional internet-based “Place Standard” survey
Source: BMC Public Health. 2022 Feb 9;22:267. doi: 10.1186/s12889-022-12706-y (PMC8830016; doi:10.1186/s12889-022-12706-y)
Supplement: Supplementary file 1 — Additional file 1. [file 12889_2022_12706_MOESM1_ESM.pdf]

Additional file 1

Greek edition of the booklet and online version of the Place Standard tool

The Place Standard tool was adapted to an online format using Google Forms (https://forms.gle/6nd2wm3aCFUDt1LF8). The full booklet version of the Greek edition of the Place Standard tool can be found in the Cyprus University of Technology repository KTISIS (https://ktisis.cut.ac.cy/handle/10488/23511). Alternatively, it can be made available upon reasonable request from the authors. It should also be available from the developers of the original Place Standard team at NHS Scotland.

For the online survey, an abridged version of the Greek translated Place Standard tool was used. This included the 14 core items along with the brief introduction for each and all prompt questions, as per the original Place Standard; however, free-comment text boxes, which appear in the full version, were not included in the online survey.

Alongside the motto "*if we don't measure it, we won't improve it*", study material featured for promotional purposes an original street art image found on a wall in old Nicosia's historic centre, depicting a café-like scene in a town square where people of all ages and backgrounds appear to have come together in a common task – see below.

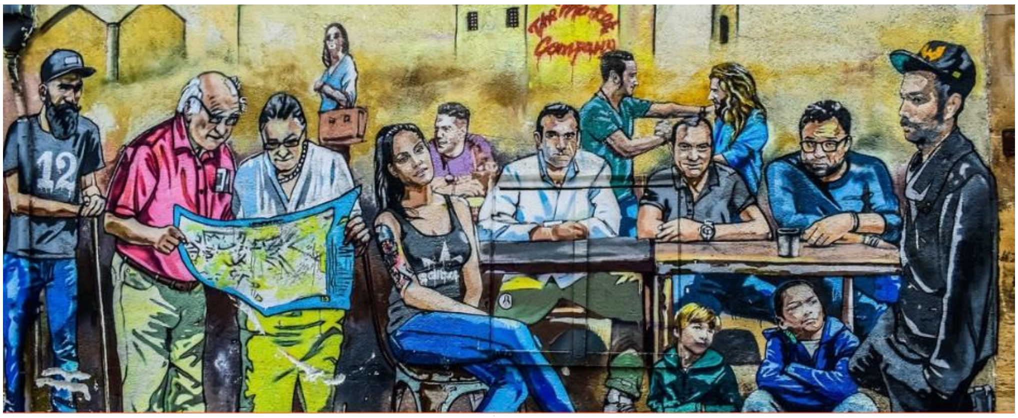

Τεχνολογικό
Πανεπιστήμιο
Κύπρου
