# Supplementary material for: Profiling the variability and inequity in the residential environment in Cyprus according to citizens’ ratings: a cross-sectional internet-based “Place Standard” survey
Source: BMC Public Health. 2022 Feb 9;22:267. doi: 10.1186/s12889-022-12706-y (PMC8830016; doi:10.1186/s12889-022-12706-y)
Supplement: Supplementary file 2 — Additional file 2. [file 12889_2022_12706_MOESM2_ESM.docx]

## **Additional file 2**

## **Table S1:** Observed differences in percentage of participants reporting that large improvements are needed across the 14 dimensions of the neighborhood’s environment assessed using the Place Standard by subjective assessment of neighborhood’s social position

|  | **Subjective assessment of neighborhood’s social position** | | | | **Steps 4-7**  **[ref: 8-10)** | **Steps 1-3**  **[ref: 8-10)** |
| --- | --- | --- | --- | --- | --- | --- |
| **Place Standard domains** | **Relatively disadvantaged (1-3)** | **Around midpoint (4-7)** | **Relatively privileged (8-10)** | **p-value** | **OR (95% CI)** | **OR (95% CI)** |
| **Q1: Moving around** | 79.8% | 50.0% | 34.7% | <0.001 | 1.9 (1.2. 2.9) | 7.4 (3.9, 14.2) |
| **Q2: Public transport** | 71.1% | 56.7% | 52.1% | 0.06 | 1.2 (0.8, 1.8) | 2.3 (1.3, 4.1) |
| **Q3: Traffic & Parking** | 75.9% | 40.4% | 29.8% | <0.001 | 1.6 (1.0, 2.5) | 7.4 (3.9, 14.1) |
| **Q4: Streets & Spaces** | 86.7% | 39.3% | 24.0% | <0.001 | 2.1 (1.3, 3.3) | 20.8 (9.7, 44.4) |
| **Q5: Natural space** | 71.1% | 38.2% | 28.9% | <0.001 | 1.5 (0.9, 2.4) | 6.0 (3.3, 11.2) |
| **Q6: Play & Recreation** | 70.2% | 39.9% | 24.8% | <0.001 | 2.0 (1.3, 3.2) | 7.2 (3.8, 13.4) |
| **Q7: Facilities & Amenities** | 61.9% | 34.4% | 19.0% | <0.001 | 2.2 (1.3, 3.7) | 6.9 (3.7, 13.0) |
| **Q8: Work & Local economy** | 52.4% | 20.0% | 21.5% | <0.001 | 0.9 (0.5, 1.5) | 4.0 (2.2, 7.4) |
| **Q9: Housing & Community** | 64.3% | 29.1% | 21.5% | <0.001 | 1.5 (0.9, 2.5) | 6.6 (3.5. 12.3) |
| **Q10: Social contact** | 66.7% | 38.1% | 41.3% | <0.001 | 0.9 (0.6, 1.3) | 2.8 (1.6, 5.1) |
| **Q11: Identity & Belonging** | 75.0% | 30.1% | 19.8% | <0.001 | 1.7 (1.0, 2.9) | 12.1 (6.2, 23.6) |
| **Q12: Feeling safe** | 56.0% | 11.5% | 6.6% | <0.001 | 1.8 (0.8, 4.1) | 17.9 (7.8, 41.4) |
| **Q13: Care & Maintenance** | 81.0% | 35.0% | 19.0% | <0.001 | 2.3 (1.4, 3.8) | 18.1 (8.9, 36.8) |
| **Q14: Influence/Sense of control** | 81.0% | 59.1% | 48.8% | <0.001 | 1.5 (1.0, 2.3) | 4.5 (2.3, 8.6) |

## **Table S2:** Observed differences in percentage of participants reporting that large improvements are needed across the 14 domains of the neighborhood’s environment by subjective assessment of neighborhood’s social position (per unit increase)

|  | **Subjective assessment of neighborhood’s social position** | | | | | | | | | | **Per 1 step increase** |
| --- | --- | --- | --- | --- | --- | --- | --- | --- | --- | --- | --- |
| **Place Standard** | **1** | **2** | **3** | **4** | **5** | **6** | **7** | **8** | **9** | **p-value** | **OR (95% CI)** |
| **Moving around** | 95.0% | 81.5% | 70.3% | 61.5% | 63.7% | 44.3% | 40.9% | 39.7% | 27.1% | <0.001 | 1.4 (1.3, 1.6) |
| **Public transport** | 65.0% | 73.1% | 73.0% | 61.5% | 65.0% | 54.4% | 50.9% | 46.6% | 60.4% | 0.30 | 1.1 (1.0, 1.2) |
| **Traffic & Parking** | 85.0% | 76.9% | 70.3% | 38.5% | 53.8% | 49.3% | 25.5% | 31.5% | 27.1% | <0.001 | 1.4 (1.3, 1.5) |
| **Streets & Spaces** | 100.0% | 92.3% | 75.7% | 57.7% | 61.3% | 31.9% | 23.6% | 24.7% | 22.9% | <0.001 | 1.7 (1.5, 1.9) |
| **Natural space** | 85.0% | 92.3% | 48.6% | 42.3% | 58.8% | 30.4% | 27.3% | 32.9% | 22.9% | <0.001 | 1.4 (1.3, 1.5) |
| **Play & Recreation** | 85.0% | 74.1% | 59.5% | 50.0% | 56.3% | 37.1% | 27.3% | 30.1% | 16.7% | <0.001 | 1.4 (1.3, 1.6) |
| **Facilities & Amenities** | 70.0% | 66.7% | 54.1% | 42.3% | 38.8% | 37.1% | 27.5% | 20.5% | 16.7% | <0.001 | 1.4 (1.2, 1.5) |
| **Work/Local economy** | 80.0% | 66.7% | 27.0% | 28.0% | 23.8% | 21.4% | 14.5% | 23.3% | 18.8% | <0.001 | 1.3 (1.2, 1.6) |
| **Housing & Community** | 70.0% | 59.3% | 64.9% | 34.6% | 31.1% | 25.7% | 28.4% | 24.7% | 16.7% | <0.001 | 1.3 (1.2, 1.5) |
| **Social contact** | 75.0% | 74.1% | 56.8% | 46.2% | 46.3% | 38.6% | 30.0% | 49.3% | 23.3% | <0.001 | 1.2 (1.1, 1.3) |
| **Identity & Belonging** | 85.0% | 74.1% | 70.3% | 34.6% | 43.8% | 30.0% | 19.1% | 19.2% | 20.8% | <0.001 | 1.5 (1.4, 1.7) |
| **Feeling safe** | 85.0% | 59.3% | 37.8% | 30.8% | 11.3% | 10.0% | 8.2% | 6.8% | 6.3% | <0.001 | 1.7 (1.5, 2.0) |
| **Care & Maintenance** | 95.0% | 92.6% | 64.9% | 69.2% | 43.8% | 31.4% | 22.7% | 19.2% | 18.8% | <0.001 | 1.7 (1.5, 1.9) |
| **Influence/Control** | 85.0% | 81.5% | 78.4% | 73.1% | 68.8% | 51.4% | 53.6% | 50.7% | 45.8% | <0.001 | 1.3 (1.2, 1.4) |
