# Supplementary material for: Profiling the variability and inequity in the residential environment in Cyprus according to citizens’ ratings: a cross-sectional internet-based “Place Standard” survey
Source: BMC Public Health. 2022 Feb 9;22:267. doi: 10.1186/s12889-022-12706-y (PMC8830016; doi:10.1186/s12889-022-12706-y)
Supplement: Supplementary file 3 — Additional file 3. [file 12889_2022_12706_MOESM3_ESM.docx]

## **Additional file 3**

## **Table S3:** Observed differences in perceived neighborhood environment domain and total scores by socio-demographic characteristics of the participants.

|  |  | **Built environment**  **(6 items)** | **Physical environment (3 items)** | **Social environment (2 items)** | **Service environment (3 items)** | **Total Score**  **(projected to 1-100)** | **Social position 10-step ladder** |
| --- | --- | --- | --- | --- | --- | --- | --- |
| **Variable†** | **Variable categories** | **Mean (SD)** | **Mean (SD)** | **Mean (SD)** | **Mean (SD)** | **Mean (SD)** | **Mean (SD)** |
| **Gender** | **Male** |  | 39.3 (26.9) | 40.6 (26.3) |  |  |  |
|  | **Female** |  | 33.2 (24.3) | 36.5 (26.8) |  |  |  |
|  | **p-value** |  | 0.01 | 0.10 |  |  |  |
| **Age** | **<35** |  | 37.9 (23.2) |  |  |  |  |
|  | **35-54** |  | 32.9 (25.2) |  |  |  |  |
|  | **55+** |  | 38.5 (28.6) |  |  |  |  |
|  | **p-value** |  | 0.07 |  |  |  |  |
| **Household size** | **Single-person** | 32.5 (18.1) |  |  |  |  |  |
|  | **At least two** | 38.2 (20.1) |  |  |  |  |  |
|  | **p-value** | 0.03 |  |  |  |  |  |
| **Educational attainment** | **Up to secondary** | 34.1 (22.8) |  |  |  | 36.3 (20.8) | 5.8 (2.3) |
|  | **Tertiary-College** | 36.0 (21.8) |  |  |  | 34.1 (20.5) | 5.0 (2.3) |
|  | **Tertiary-University** | 34.8 (18.8) |  |  |  | 34.5 (16.7) | 5.6 (2.2) |
|  | **Postgraduate degree** | 38.6 (19.0) |  |  |  | 37.9 (17.3) | 6.1 (2.0) |
|  | **Doctoral degree** | 45.1 (21.1) |  |  |  | 41.9 (18.3) | 6.8 (1.8) |
|  | **p-value** | 0.022 |  |  |  | 0.10 | <0.001 |
| **Employment status** | **Employed** |  |  |  | 37.1 (19.9) | 37.2 (17.9) |  |
|  | **Not econ. active/ Retired** |  |  |  | 36.7 (20.1) | 36.7 (17.7) |  |
|  | **Unemployment** |  |  |  | 20.6 (21.2) | 26.1 (16.9) |  |
|  | **p-value** |  |  |  | 0.004 | 0.04 |  |
| **Financial difficulties** | **No** | 40.1 (19.2) | 37.4 (25.2) |  | 38.3 (19.4) | 38.8 (17.2) | 6.1 (2.1) |
|  | **Yes** | 32.9 (20.5) | 32.1 (25.5) |  | 33.3 (21.2) | 33.2 (18.8) | 5.6 (2.3) |
|  | **p-value** | <0.001 | 0.03 |  | 0.01 | 0.001 | 0.009 |
| **House tenure** | **Owner-occupied** | 39.0 (19.8) |  |  | 37.4 (20.1) | 37.8 (17.5) | 6.0 (2.2) |
|  | **Not owner occupied** | 32.7 (19.7) |  |  | 33.8 (20.3) | 33.7 (19.0) | 5.6 (2.2) |
|  | **p-value** | 0.003 |  |  | 0.09 | 0.03 | 0.08 |
| **House type** | **House** | 39.2 (20.9) |  |  |  |  |  |
|  | **Apartment** | 33.9 (17.4) |  |  |  |  |  |
|  | **p-value** | 0.006 |  |  |  |  |  |
| **†** To facilitate interpretation, only variables for which a statistically significant association at the 10% level with at least one of the neighbourhood environment scores are presented in the Table | | | | | | | |
